# Increased granulovacuolar degeneration in the thalamus and higher neurofibrillary tangle Braak stages in bipolar disorder

**DOI:** 10.1111/pcn.13891

**Published:** 2025-09-01

**Authors:** Akito Nagakura, Ito Kawakami, Araki Kimura, Kenji Ikeda, Kenichi Oshima, Mie Kubota‐Sakashita, Tadafumi Kato

**Affiliations:** ^1^ Tokyo Metropolitan Matsuzawa Hospital Tokyo Japan; ^2^ Department of Psychiatry and Behavioral Science Juntendo University Graduate School of Medicine Tokyo Japan; ^3^ Molecular Pathology and Histology Tokyo Metropolitan Institute of Medical Sciences Tokyo Japan; ^4^ Dementia Research Project Tokyo Metropolitan Institute of Medical Science Tokyo Japan

**Keywords:** bipolar disorder, granulovacuolar degeneration, neurodegenerative protein, neuropathology, paraventricular thalamic nucleus

## Abstract

**Aim:**

Recent neuropathological studies suggest that the accumulation of neurodegenerative disease–associated proteins in subcortical structures may contribute to mood symptoms. Animal models have highlighted the role of the paraventricular thalamic nucleus (PVT) in bipolar disorder (BD) pathophysiology. However, neuropathological investigations in the thalamus in BD remain limited. This study aimed to examine neurodegenerative pathology in the thalamus and medial temporal region including the hippocampus in patients with BD.

**Methods:**

Postmortem brain tissues of the thalamus and medial temporal region of nine patients with BD and nine age‐matched controls were obtained from Matsuzawa Hospital, with additional medial temporal samples of 14 BD cases acquired from the Stanley Foundation Brain Bank. Immunohistochemical analyses were performed using antibodies against phosphorylated tau, amyloid‐*β*, *α*‐synuclein, TDP‐43, and granulovacuolar degeneration (GVD) markers including CHMP2B and CK‐1*δ*.

**Results:**

The 23 BD cases exhibited a significantly greater burden of tau pathologies, including higher neurofibrillary tangle Braak stages (*P* = 0.015) and more severe argyrophilic grain Saito stage (*P* = 0.029), compared with the nine controls. Notably, CHMP2B‐positive GVD was significantly more frequently observed in the PVT of BD cases than in the controls (five of nine vs. zero of nine, *P* = 0.029).

**Conclusions:**

These findings suggest that neurodegenerative processes, particularly tau pathology and CHMP2B‐positive GVD in the PVT may play a role in BD pathophysiology.

Bipolar disorder (BD) is a severe mood disorder characterized by recurrent episodes of depression and mania. The first mood episode typically occurs in adolescence or early adulthood, although onset can occur in middle or older age, making it a significant burden across multiple life stages.^1^, ^2^ Despite extensive research, the biological underpinnings of BD remain incompletely understood.^3^

It has become increasingly recognized that mood and psychotic symptoms in older adults may be linked to underlying organic neurodegenerative pathology.^4^, ^5^, ^6^ Recent neuropathological studies in patients with neurodegenerative disorders or in the general population have shown that accumulation of neurodegenerative disease–associated proteins in subcortical areas such as the nucleus accumbens, substantia nigra, ventral tegmental area, and locus coeruleus is associated with mood symptoms and psychotic symptoms such as hallucinations and delusions.^7^, ^8^, ^9^, ^10^ Clinically, patients with dementia can exhibit BD‐like symptoms, including irritability, elevated mood, and depression.^11^ These symptoms may appear as prodromal features before cognitive impairment becomes apparent,^4^ and, for example, research has shown that 10% of patients with behavioral variant frontotemporal dementia had prior clinical diagnoses of BD.^12^

Patients with BD have a higher risk of dementia, with an odds ratio of 3.0,^13^ and genome‐wide association studies have revealed significant genetic correlations between BD and Alzheimer disease (AD).^14^ Numerous studies have documented evidence of enhanced inflammatory processes and oxidative stress in BD,^15^, ^16^ which can play crucial roles in the neurodegenerative protein accumulation.^17^ These findings suggest shared genetic and molecular pathophysiological mechanisms between BD and neurodegenerative diseases.^18^

However, studies examining neurodegenerative protein accumulation in postmortem BD brains are limited. While some reports indicate the presence of AD pathology in a subset of cases,^19^ others have found no significant increase in AD pathology in patients with BD,^20^ leading to inconsistent results. A recent postmortem study reported argyrophilic grain accumulation in all 11 BD cases examined.^21^

To investigate the effects of neurodegeneration, we focused on the hippocampus and the thalamus. The hippocampus shows the early effects of neurodegeneration, including tau pathology, and possesses dense neural connections with the cerebral cortex and subcortical regions such as the thalamus, amygdala, and nucleus accumbens.^22^, ^23^ Considering the genetic correlation between dementia and BD,^24^ we regarded the hippocampus as an important region for assessing neurodegenerative pathology in BD. Furthermore, within the thalamus, the paraventricular thalamic nucleus (PVT) is increasingly recognized for its crucial role in the emotion regulations.^3^, ^25^ In a BD animal model using Polg1 mutant transgenic mice, abnormal mitochondrial hotspots were identified in the PVT, suggesting that structural and functional abnormalities in this region play a crucial role in BD symptom manifestation.^26^ The ENIGMA (Enhancing Neuroimaging Genetics Through Meta‐Analysis) consortium, a large‐scale international neuroimaging initiative, has also demonstrated volumetric abnormalities in subcortical regions, specifically the hippocampus and the thalamus.^27^

The present study aims to examine the role of these neurodegenerative processes in the pathophysiology of BD by conducting systematic neuropathological analyses of postmortem brain tissues from patients with BD. We focused specifically on two regions: (i) the medial temporal lobe, including the hippocampus; and (ii) the thalamus, including the PVT, to investigate the accumulation of major neurodegenerative disease–associated proteins, phosphorylated tau, amyloid‐*β*, *α*‐synuclein, and TDP‐43.

## Methods

### Patients

We examined postmortem tissues of the thalamus and the medial temporal region including hippocampus of nine BD cases and nine controls from Matsuzawa Hospital, and the medial temporal region including the hippocampus of 14 BD cases from the Stanley Foundation Brain Bank. For the Matsuzawa Hospital cases, BD diagnosis was made according to DSM‐5 through retrospective analysis of medical records. Detailed clinical information for the Stanley Foundation Brain Collection samples was summarized elsewhere.^28^ Controls who had no history of neurological or psychiatric disorders as confirmed by review of the medical records and interviews by psychiatrists were obtained from Matsuzawa Hospital. Both cases with BD and controls had no clinical diagnosis of neurodegenerative diseases including dementia. The demographic data of all patients are shown in Table 1.

**Table 1:** Clinical and neuropathological characteristics of individual BD cases from Matsuzawa Hospital

Case	Early/late	Age at onset, y	Age at death, y	NFT Braak stage	GVD in the thalamus
1	Early	19	77	3	−
2	Early	26	75	3	+
3	Early	30	56	1	−
4	Early	30	62	0	−
5	Early	30	63	2	−
6	Late	44	47	0	+
7	Late	46	76	4	+
8	Late	59	59	2	+
9	Late	59	77	3	+

BD, bipolar disorder; GVD, granulovacuolar degeneration; NFT, neurofibrillary tangle.

To examine the differences in BD neuropathology based on the age at onset, the nine cases from Matsuzawa Hospital whose medical records were available were subdivided into the early‐ and late‐onset groups, although this comparison is preliminary because of the small number of samples in each group. While there is no clear consensus on the age threshold for stratifying BD by age at onset, cutoff ages of 40 or 50 years are commonly used,^29^, ^30^ with late‐onset cases suggested to represent a distinct disease subtype. Following the recommendations of the 2015 task force report,^31^ we adopted 40 years as the cutoff age.

### Tissue processing and histological staining

Brains were fixed in 10% neutral buffered formalin for at least 2 weeks at room temperature, embedded in paraffin, and sectioned at 6‐ to 7‐μm thickness. The sections were stained with hematoxylin–eosin (HE) and Klüver–Barrera stains. Selected regions were additionally stained with the modified Gallyas–Braak silver method and the methenamine silver stain.

### Immunohistochemistry

Immunohistochemical staining for neurodegenerative disease–associated proteins was performed on the medial temporal sections including the hippocampus using the following primary antibodies: anti–phospho‐tau (AT8, mouse monoclonal, 1:10000; Invitrogen, cat# MN1020), anti–*β*‐Amyloid (4G8, mouse monoclonal, 1:1000; BioLegend, cat# SIG‐39200), anti *α*‐synuclein (pSyn, rabbit monoclonal, 1:1000; Abcam, cat# ab51253), and anti–TDP‐43 (TDP‐43, mouse‐monoclonal, 1:10000; Cosmo‐bio, cat# 66318‐1‐Ig). For granulovacuolar degeneration (GVD) assessment, hippocampal and thalamic sections were immunostained with anti‐CHMP2B (rabbit polyclonal, 1:2000; Abcam, cat# ab33174) and anti‐Casein Kinase 1*δ* (rabbit polyclonal, 1:10000; Proteintech, cat# 14388‐1‐AP). Anti‐calretinin antibody (rabbit polyclonal, 1:100; Abcam, cat# ab16694) was used to identify the PVT.

For immunohistochemistry, sections were deparaffinized through xylene and ethanol. Heat‐mediated antigen retrieval was performed by autoclaving sections in 10 mM of citrate‐buffered saline (pH 6.0) at 121°C for 10 min. Endogenous peroxidase activity was blocked with 0.3% hydrogen peroxide in phosphate‐buffered saline. After blocking with 10% goat serum in 0.01M phosphate‐buffered saline containing 0.1% triton X‐100, sections were incubated with primary antibodies overnight at 4°C. Immunoreactivity was visualized using a polymer‐based detection method with goat anti‐rabbit IgG H + L (horseradish peroxidase polymer) (Abcam) as the secondary antibody applied for 30 min at room temperature, followed by 3,3′‐diaminobenzidine as chromogen. Known positive and negative controls, such as sections from patients with AD or dementia with Lewy bodies (DLB) and young controls without any neuropsychiatric disorders, were included in each staining run.

### Assessment of histopathology

Neurofibrillary tangle (NFT) staging was assessed according to the Braak criteria (stages 0–VI) using AT8 immunostaining.^32^ Amyloid‐*β* plaque distribution was assessed according to Thal phases (0–5) using A*β* immunostaining.^33^ Pathological AD diagnosis followed the National Institute on Aging‐Alzheimer's Association (NIA‐AA) guidelines.^34^ Argyrophilic grain distribution was evaluated according to Saito staging (stages 0–III)^35^ using AT8 immunostaining. Lewy‐related pathology was assessed following the fourth consensus report of the DLB consortium using *α*‐synuclein immunostaining.^36^

We first performed immunohistochemical staining on sections of the medial temporal lobe including hippocampus from both Stanley Foundation and Matsuzawa Hospital cases. For the Stanley Foundation cases, which showed only early neurodegenerative changes, staging was determined using medial temporal lobe sections. For Matsuzawa Hospital cases, which showed more advanced tau and amyloid‐*β* pathology, additional sections from the frontal, parietal and occipital lobes, basal forebrain, cerebellum, thalamus, and striatum were examined when necessary for accurate staging.

### Assessment of GVD

In several of the nine cases from Matsuzawa Hospital, HE staining revealed GVD‐like lesions in the medial thalamus, predominantly in the PVT. GVD is a structure observed in neuronal cytoplasm, characterized by membrane‐bound clear vacuoles observed in neuronal cytoplasm with dense core granules in their centers. To identify whether these structures represent GVD and characterize their features, immunohistochemical analysis was performed and compared with age‐matched controls. We used CHMP2B and CK‐1*δ* antibodies, which are reported to be specific markers for GVD.^37^, ^38^ Previous studies have demonstrated that these primary antibodies can identify GVD by labeling the granular components within these structures.^39^, ^40^

### Statistical analysis

Demographic variables were compared between groups using the Student *t* test for continuous variables and the *χ* ^2^ test for categorical variables. For variables with nonnormal distribution, the Mann–Whitney *U* test was applied. Fisher exact test was used when comparing the presence or absence of pathological findings between groups due to small sample sizes. The threshold for statistical significance was set at *P* < 0.05. All statistical analyses were performed using SPSS version 27.0 (IBM).

This study was approved by the ethics committees of Matsuzawa Hospital (ethics approval number: 2022‐3) and Juntendo University (study number: M19‐0278) and conducted in accordance with the ethical principles of the Declaration of Helsinki. Written informed consent was obtained from the patients' proxies, and their anonymity was preserved.

## Results

### Neurodegenerative staging in BD cases and controls

**Fig. 1:**
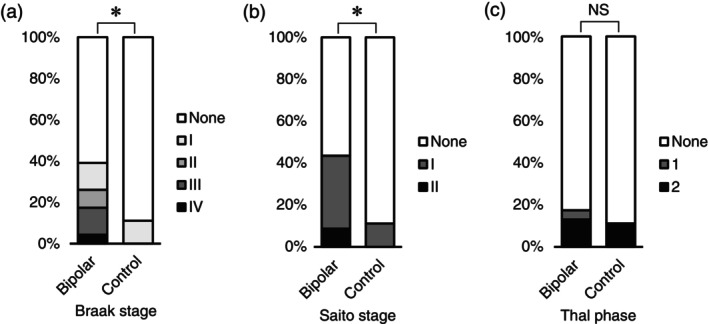
Comparison of neurodegenerative pathology between patients with bipolar disorder (BD) and controls. Neurodegenerative pathological changes were compared between patients with BD (*n* = 23) and age‐matched controls (*n* = 9). (a) Patients with BD exhibited significantly higher neurofibrillary tangle stage (Braak stage) compared with controls (*P* = 0.015, two‐tailed *t* test). (b) Patients with BD showed significantly higher Saito stage for argyrophilic grains compared with controls (*P* = 0.029). (c) There are no significant differences in Thal phases for amyloid‐*β* plaques. The y‐axis denotes the percentage among cases or controls. *P<0.05.

The neurodegenerative staging of 23 BD cases and nine age‐matched controls is presented in Figure 1. The mean age at onset in the BD group was 28.2 years (SD 13.6 years). The mean age at death was 51.3 years (SD 16.5 years) in the BD group and 48.1 years (SD 6.9 years) in the control group, with no significant difference between the two groups.

NFT accumulation was observed in 39.1% (nine of 23 cases) of BD cases, while only 11.1% (one of nine cases) of controls showed such pathology. Assessment of tau pathology using Braak staging revealed significantly higher stages in the BD group compared with the control group (*P* = 0.015, two‐tailed *t* test).

Argyrophilic grain pathology of Saito stage I or higher was observed in 10 cases (43.5%) in the BD group, but in only one case of nine controls (11.1%; *P* = 0.12, Fisher exact test). When comparing the severity of the stages, the BD group showed significantly higher Saito stages than the controls (*P* = 0.029, two‐tailed *t* test). All these tau pathologies, including both NFTs and argyrophilic grains, remained confined to the medial temporal lobe and did not propagate to the other neocortical regions.

Amyloid‐*β* pathology was observed in 17.4% of the BD cases and 11.1% of the controls, showing comparable levels of accumulation. Neither *α*‐synuclein nor TDP‐43 showed significant accumulation in either group.

### Late‐onset BD and neurodegenerative changes

**Fig. 2:**
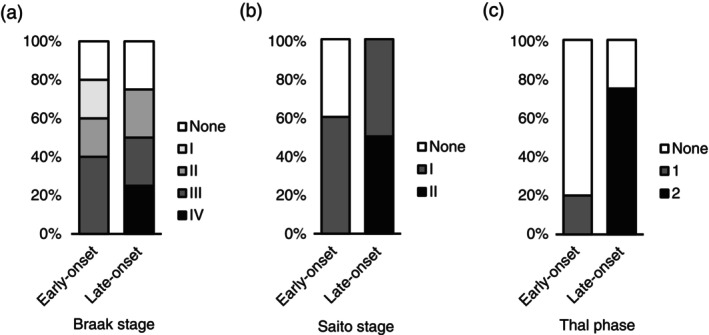
Comparison of neurodegenerative pathology between early‐onset and late‐onset bipolar disorder (BD). Neurodegenerative pathological changes were compared between early‐onset (age at onset <40 years) and late‐onset (age at onset ≥40 years) BD cases. (a, b) There are no significant differences in the neurofibrillary tangle stage (Braak stage) (a) and the Saito stage for argyrophilic grains (b) between the early‐ and late‐onset groups. (c) The late‐onset group shows significantly higher Thal phases for amyloid‐*β* plaque compared with the early‐onset group (*P* = 0.048, Fisher exact test). The y‐axis represents the percentage of cases in each stage/phase. NS, not significant.

We stratified the Matsuzawa Hospital BD cases into early‐ and late‐onset groups using an age at onset of 40 years as the cutoff, although the comparison is preliminary because of the small number of samples in each group. The early‐onset group (*n* = 5) had a mean age at onset of 27 years, while the late‐onset group (*n* = 4) had a mean age at onset of 52 years. The mean age at death was similar between the two subgroups: 66.6 years for the early‐onset group and 65.8 years for the late‐onset group, with no significant difference between the groups. Individual neurodegenerative changes are shown in Figure 2. NFT accumulation was observed at similar frequencies between groups, but the late‐onset group showed more severe pathology, with three of four cases showing Braak stage ≥II. All four late‐onset cases exhibited argyrophilic grains, with two cases showing Saito stage ≥II. Amyloid‐*β* accumulation was present in three of four late‐onset cases, which was statistically significantly more common compared with the early‐onset cases (*P* = 0.048, Fisher exact test).

### GVD in the thalamus

**Fig. 3:**
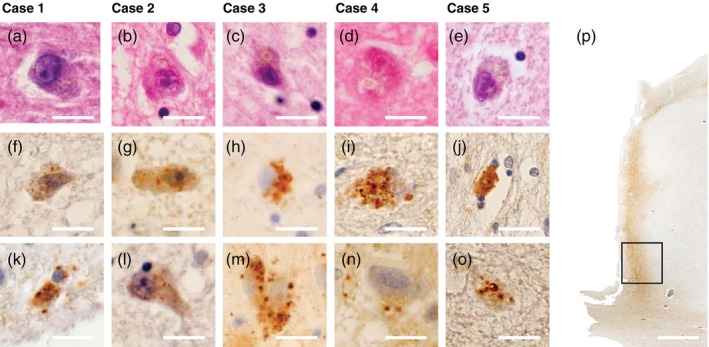
CHMP2B‐positive granulovacuolar degeneration (GVD) in the thalamus of patients with bipolar disorder (BD). All cases are patients with BD from Matsuzawa Hospital. (a–e) Hematoxylin–eosin staining showing structures in neuronal cytoplasm with vacuoles containing single or multiple granular cores. (f–o) CHMP2B immunostaining, commonly used for GVD identification, demonstrating granular staining in neuronal cytoplasm. (p) Calretinin immunostaining of case 2's thalamus for identification of the paraventricular thalamic nucleus (PVT). Calretinin is known to specifically label PVT in the thalamus, showing the PVT as a band‐like structure along the lateral ventricle. The black square indicates the region where GVD was observed in case 2 (b, g, l). In other cases, GVD was similarly observed in the medial thalamus along the lateral ventricle. Scale bar = 10 μm (a–o), scale bar = 1 mm (p).

In our preliminary analysis of thalamic samples using HE staining, we noted that some BD cases show GVD‐like findings. To further verify this finding, we performed immunostaining with both CHMP‐2B and CK‐1*δ*, which are standard markers for detecting GVD. While none of the cases in either the BD or control groups showed positive staining for CK‐1*δ*, CHMP2B immunostaining revealed granular cytoplasmic staining in neurons in five of nine BD cases, which was significantly higher than in controls (*P* = 0.029, Fisher exact test). The individual case characteristics are presented in Table 1. Furthermore, stratification of GVD in the PVT by age of onset showed that while GVD was positive in only one of five early‐onset BD cases, all four late‐onset BD cases were positive (*P* = 0.048, Fisher exact test). There was no statistically significant difference in the age at death between these two subgroups. Although faint staining was observed in glial cells in only a few control cases, no control cases showed granular cytoplasmic staining in neurons. HE and CHMP2B staining images for each case are shown in Figure 3. To locate the CHMP2B‐positive GVD in the thalamus, the adjacent sections were stained with anti‐calretinin antibody, because calretinin is a marker of the PVT.^41^ We found that the regions where GVD was observed were overlapped with the PVT regions shown by calretinin‐positive immunoreactivity (Fig. 3p).

In the hippocampus, which represents the initial and most common site of GVD appearance in typical neurodegenerative cases, GVD was present in four of nine BD cases. All of these four cases also showed CHMP2B‐positive GVD in the thalamus. In contrast, among the control group, only one of nine cases (11.1%) showed GVD in the hippocampus, and this case did not exhibit any GVD pathology in the thalamus.

## Discussion

The present study examined neuropathological findings in postmortem brains from patients with BD, focusing on neurodegenerative disease–associated protein accumulation in the thalamus and medial temporal region including hippocampus. We revealed that: (i) NFT stages were higher in patients with BD compared with controls, (ii) late‐onset BD cases showed a higher prevalence of AD‐type pathology compared with early‐onset BD cases, and (iii) presence of CHMP2B‐positive GVD in the PVT in patients with BD.

### NFT and A*β* plaque pathology in BD

Our study demonstrated a significantly higher NFT Braak stage in BD cases compared with controls. While NFT pathology in BD cases did not extend to the neocortical stages (Braak stages V/VI), a subset of BD cases showed progression beyond the entorhinal region (stages I/II) into limbic areas (stages III/IV). Such progression into the limbic stages was not observed in our control group. Regarding amyloid‐*β* (A*β*) pathology, the overall prevalence did not significantly differ between the BD (17.4%) and control (11.1%) groups. Among our 23 BD cases, diffuse plaques constituted the majority of A*β* pathology, with neuritic plaques observed in only one late‐onset case. Given that diffuse plaques constituted the majority of the A*β* pathology in our BD cohort, the observed NFT accumulation may correspond to primary age‐related tauopathy (PART).^42^ PART is a neuropathological condition characterized by NFTs confined primarily to the entorhinal cortex and limbic areas, with absent or only sparse A*β* plaque deposition (typically Thal phase 0–2). While not all BD cases exhibited NFT accumulation, the significantly higher prevalence of this pathology and its more extended distribution in BD cases compared with controls suggest that BD may be associated with vulnerability to the earlier accumulation of NFTs or accelerated progression into limbic areas.^43^, ^44^

### AGD pathology in BD

We observed argyrophilic grain pathology in 43.5% of BD cases. While this indicates substantial presence, our prevalence rate differs from the findings of Shioya et al.^21^ They reported argyrophilic grain pathology in all 11 BD cases examined. This discrepancy may be attributed to two factors: they included very mild (stage 0.5) grain accumulation in their assessment, and the age of onset was higher in their study (mean 41.8 years) compared with the current study (mean 28.2 years). Consistent with the findings of Shioya et al., all four cases in the late‐onset group (age of onset ≥40 years) in the current study showed argyrophilic grain accumulation of Saito stage ≥I, suggesting that the prevalence of grain pathology may indeed be associated with age of onset.

### Lewy body disease pathology

In the present study, no significant Lewy body disease pathology was observed in either the BD group or the control group. However, DLB is known to have a higher prevalence of depression compared with other dementia types, with depressive symptoms often preceding the onset of motor and cognitive manifestations in patients with DLB.^45^, ^46^ Recently, prodromal DLB has been classified into three subtypes based on initial presentation: delirium onset, psychiatric onset, and mild cognitive impairment onset.^47^ Manic episodes have been reported as potential prodromal symptoms of DLB.^48^, ^49^ However, a large autopsy study of 1640 DLB cases suggests that the frequency of such psychiatric presentations may not be particularly high in the overall DLB population.^50^ In our study, the limited number of late‐onset cases (*n* = 4) may have precluded the detection of significant associations with Lewy body disease pathology.

### Late‐onset BD and neurodegenerative changes

Stratification of cases using a 40‐year age‐at‐onset cutoff showed that late‐onset cases had significantly more A*β* deposition (Thal phases, *P* = 0.048) than early‐onset cases, although the number of patients in each group was too small to draw a definite conclusion. One late‐onset case exhibited neuritic plaques, while diffuse plaques were common in most BD cases. Late‐onset cases also tended to have higher NFT Braak stages, although this was not statistically significant. None of the cases in our study had been diagnosed with clinical dementia. These findings can be interpreted in two ways: first, the clinical symptoms meeting BD diagnostic criteria, particularly in individuals with late‐onset cases, could represent manifestations of underlying neurodegenerative processes, such as the PART pathology we observed.^11^, ^18^, ^51^ This interpretation can be related to the concept of mild behavioral impairment (MBI). MBI encompasses five domains, including changes in affect, depression, and mood lability.^52^ It has recently been recognized as an early sign of dementia, and the PART‐like pathology observed in our late‐onset BD group suggests that late‐onset BD could be contextualized within MBI.^4^, ^53^ However, it is important to note that these two apparently different interpretations (late‐onset BD is caused by neurodegenerative processes, and the observed symptoms represent prodromal manifestations of early‐stage dementia) describe the same clinical phenomena from a different perspective. Importantly, current diagnostic criteria for mental disorders are based largely on behavioral symptoms assessed through clinical interviews and are not yet grounded in neuropathological evidence. Therefore, future research should aim to bridge this gap by establishing direct correspondence between psychiatric diagnoses and underlying neuropathological findings.

It is noteworthy that tau pathology begins to appear in the brainstem, particularly in the locus coeruleus, during the teen years and can progress to the entorhinal cortex and hippocampus as early as the 20s in some cases.^54^ Therefore, early tau pathology might play a role in BD pathophysiology even in early‐onset cases. However, since tau pathology was not observed in all late‐onset cases, it is likely that multiple factors, including other neurodegenerative disease–associated proteins and physiological aging processes, work in concert rather than tau pathology alone. These findings add to the growing evidence that late‐onset BD may be more closely linked to neurodegenerative changes compared with early‐onset BD, as previously suggested by several studies.^30^, ^31^, ^55^ This link was further supported by a recent multimodal study. Kurose et al., combining in vivo positron emission tomography imaging using ^18^F‐florzolotau with postmortem analysis, reported that individuals with late‐life mood disorders including BD exhibit a high prevalence of diverse tauopathies, suggesting that tau pathologies could be part of the underlying pathophysiological mechanisms.^56^

### CHMP2B‐positive GVD in the thalamus

In this study, we first observed CHMP2B‐positive GVD in the medial thalamus, particularly in the PVT, in approximately half of the BD cases. GVD appears in neuronal cytoplasm and is characterized by clear vacuoles with distinct membrane boundaries containing granular cores. These granular structures contain various proteins related to cytoskeletal organization, autophagy, and cellular stress.^57^, ^58^ Typically, GVD emerges and spreads according to a specific staging pattern, beginning in the entorhinal cortex and hippocampus.^40^ Our finding that BD cases showed increased GVD in the hippocampus, a region where GVD is typically observed, compared with control cases, might suggest accelerated aging processes throughout the brain in BD.^43^, ^59^ However, the thalamus is not typically associated with GVD pathology according to the established staging scheme.^40^ Notably, a control case with hippocampal GVD did not exhibit thalamic GVD, whereas a BD case showed GVD in the thalamus but not in the hippocampus. This suggests that GVD in the thalamus might be specific to BD.

GVD has been associated with late‐stage autophagy/endocytosis markers and is considered a type of lysosome‐like structure with protein degradation activity.^37^, ^60^ While strongly associated with tau pathology,^40^ GVD is also observed in other neurodegenerative conditions, suggesting that it reflects general neurodegenerative processes and lysosomal stress rather than specific protein accumulation.^61^

The CHMP2B positivity we observed is particularly interesting, as CHMP2B is a subunit of ESCRT‐III (endosomal sorting complex required for transport III) and a component of late endosomes. While our GVD structures were CHMP2B‐positive, they were CK‐1*δ*‐negative, similar to findings reported in tau transgenic mice.^62^ This immunological profile may indicate an immature stage of GVD formation, potentially reflecting a phase when these structures are approaching fusion with lysosomes.^62^ A proteomics study of GVD showed the involvement of TOMM23, a cytosolic protein that plays a role in mitochondrial protein import.^63^ This might suggest that GVD can be either a trigger or a consequence of mitochondrial dysfunction that is postulated in BD.^3^

The PVT, where we observed CHMP2B‐positive GVD, is classified as a part of the epithalamus, with extensive connections to the anterior cingulate cortex, the insular cortex, the nucleus accumbens, and the amygdala.^64^, ^65^ This nucleus plays crucial roles in regulation of emotion,^66^, ^67^ which is often disturbed in BD. Because neural circuit manipulations of the PVT cause recurrent depression‐like episodes in mice,^68^ pathological changes in this region may play a crucial role in the pathophysiology of BD.

## Limitations

Several limitations should be considered when interpreting our findings. First, the number of patients was too small to make a definite conclusion. In particular, the comparison of early‐onset and late‐onset cases was made in very small patient populations, and the finding should be replicated in a large cohort before conclusion. Second, limited clinical information prevented detailed assessment of cognitive function and psychiatric symptoms in cases with significant neurodegenerative changes, although we confirmed the absence of clinical dementia diagnoses. Third, it was difficult to localize the PVT, where CHMP2B‐positive GVD was observed, because there are only a few studies reporting the location and shape of this nucleus in humans.^41^ Thus, it cannot be completely ruled out that interindividual variability in sectioning might have affected the results. Although anterior and posterior regions of the PVT serve different functions in rodents,^65^, ^67^ it is not known whether the human PVT can be divided into two parts. Further studies should ensure more detailed anatomical characterization.

## Conclusion

Our study demonstrated increased NFT stages in patients with BD and presence of CHMP2B‐positive GVD in the PVT in patients with BD. These findings highlight the importance of further investigation into the role of neurodegenerative protein accumulation and PVT dysfunction in BD pathophysiology.

## Disclosure statement

T.K. is an Editor‐in‐Chief of *Psychiatry and Clinical Neurosciences* but was not involved in the handling of this manuscript. Other authors declare no conflicts of interest directly related to this work.

## Author contributions

A.N., I.K. and T.K. conceived the study design. A.N., A.K. and I.K. performed the neuropathological analyses. K.I. and M.S‐K. supervised the neuropathological analyses. K.O. managed the brain samples. A.N. wrote the original draft. Other authors revised the original draft. I.K. and T.K. jointly managed the project. All the authors checked the final manuscript.
